# Interrelationship of Posttraumatic Stress, Hassles, Uplifts, and Coping in Women With a History of Severe Sexual Abuse: A Cross-Sectional Study

**DOI:** 10.1177/0886260520935479

**Published:** 2020-07-08

**Authors:** Marianne Torp Stensvehagen, Berit Arnesveen Bronken, Lars Lien, Gerry Larsson

**Affiliations:** 1 Inland Norway University of Applied Sciences, Elverum, Norway; 2 University of Oslo, Norway; 3 Innlandet Hospital Trust, Brumunddal, Norway; 4 Swedish Defence University, Stockholm, Sweden

**Keywords:** sexual assault, PTSD, domestic violence, sexual abuse, child abuse

## Abstract

Experiencing trauma, such as sexual abuse, increases the risk of a negative health outcome. The aim of the present study was to compare two groups of female survivors of sexual abuse, one group with a lower indication of posttraumatic stress disorder (L-PTSD) and one with a higher indication of posttraumatic stress disorder (H-PTSD). We hypothesized that, with a history of sexual abuse, higher levels of PTSD symptoms would be associated with more daily hassles, fewer daily uplifts, and more maladaptive coping strategies, and that there would be more reporting of severe types of sexual victimization, less resourceful socioeconomic conditions and a lower level of emotional stability. A questionnaire, including measures of socioeconomic conditions, trauma experience, emotional stability (the Single-Item Measures of Personality), Posttraumatic Stress Disorder Checklist (PCL), daily hassles and uplifts (the Stress Profile), and coping strategies (the Brief Coping Orientation to Problems Experienced [COPE] questionnaire), was completed by 57 female users at nine support centers for survivors of incest and sexual abuse in Norway. The results show that the H-PTSD group reported significantly more daily hassles, fewer daily uplifts, and more use of maladaptive coping strategies. The L-PTSD group reported more emotional stability, fewer daily hassles, and more uplifts, and used more adaptive coping strategies. However, few differences were found between the H-PTSD and the L-PTSD groups with regard to severity of sexual abuse and socioeconomic conditions. The results on the hassle, uplift, and coping scales are potentially interesting from an interventional point of view. Major life events such as sexual abuse may be out of control for the afflicted victim. Appraisal of and coping with everyday events, however, can be affected and offer interesting possibilities for interventions directed at the survivor, her significant others, and professional helpers.

## Introduction

Sexual abuse is a serious traumatic experience that can lead to physical and psychological health consequences (Jina & Thomas, 2013). Sexual abuse can be defined as any sexual act to which the victim has not consented, or has been pressured or manipulated to take part (Steine et al., 2012). Worldwide, almost 35% of women have experienced physical and/or sexual violence by an intimate partner or sexual violence by a stranger (World Health Organization, 2019). In Norway, 9.4% of women report to been subjected to sexual assault as adults and 4% have experienced some form of sexual abuse before the age of 13 years (Thoresen & Hjemdal, 2014). Wamser-Nanney et al. (2017) found that experiencing sexual trauma was more strongly related to posttraumatic stress disorder (PTSD) than the death of a loved one, suggesting the importance of trauma type in understanding event centrality and adverse outcomes. Chang et al. (2017) found that both negative life events and sexual abuse victimization uniquely predicted both positive and negative outcomes, including life satisfaction, negative affect, depressive symptoms, hopelessness, and suicidal behaviors.

PTSD is characterized by re-experiencing the trauma, avoidance of stimuli that are trauma related, and negative alterations of cognition, mood arousal, and reactivity after exposure to a stressor (American Psychiatric Association [APA], 1994). Higher rates of health care contact for physical illness, lower self-esteem, and lower life satisfaction are also reported (Dugal et al., 2016; Fergusson et al., 2013; Gekker et al., 2018). A substantial proportion of individuals report interpersonal trauma, and health professionals are likely to encounter patients with this kind of trauma history. Knowledge about how these experiences can influence psychological and interpersonal functioning is therefore important (Dugal et al., 2016).

Research on stress and health took a new direction in the 1980s. It shifted from a focus on major life events to a growing body of research suggesting that smaller everyday events or stressors caused by the event affect wellbeing and physical health more than the event itself (DeLongis et al., 1982; Larsson et al., 2017; Serido et al., 2004). Hassles are demands and conditions in everyday life that are perceived as irritating, frustrating, or stressful (Kanner et al., 1981; Stefanek et al., 2012). Daily hassles experienced by female survivors of sexual abuse may include triggers (smell or sounds linked to memories from the abuse), flashbacks (fragments of memories), guilt, shame, and lack of energy or issues relating to their own bodies (not liking, not relating to, or self-harming their bodies) (Stensvehagen et al., 2019). The frequency and perceived severity of daily hassles are more strongly associated with, or better predictors of, psychopathological symptoms than major life events (Kanner et al., 1981; Lazarus, 1999, 2006).

A counterpart to daily hassles is daily uplifts. These uplifts can help in the process of coping with stress by serving as “breathers” from regular stressful encounters, sustainers of coping activity, and restorers in the recovery from harm and loss (Kanner et al., 1981; Lazarus, 1999, 2006). Uplifts experienced by adult female survivors of sexual abuse may include finding a safe place, escaping triggers, or having the energy for activities that were formerly enjoyed (Stensvehagen et al., 2019).

The effect of hassles, uplifts, and coping strategies in everyday life after the experience of sexual abuse is not well understood. One study investigated the cumulative impact of sexual abuse in childhood and adult interpersonal violence on depressive symptoms in a nonclinical sample of women (McGuigan & Middlemiss, 2005). The results indicated that women who reported greater stress due to daily hassles reported more depressive symptoms. In a review of the interrelationship of childhood physical abuse, sexual abuse, and adult health problems, Sachs-Ericsson et al. (2009) found that current life stressors mediate the relationship between abuse and health problems, and that stress exacerbates health problems in survivors of abuse.

The impact of socioeconomic conditions must also be considered, because strong connections link the experience of trauma with adult health and adult socioeconomic status (Conroy et al., 2010; Fergusson et al., 2013). Font and Maguire-Jack (2016) found that respondents reporting early adverse experiences had a significantly higher probability of dropping out of high school and being either divorced or separated, and a lower probability of having a college degree or being married or widowed.

Reactions under stress cannot be predicted without reference to personality traits and processes that account for the individual differences in the ways in which people respond to a stressful stimulus (Lazarus, 1999, 2006). Personality trait theory often uses five dimensions to describe personality: extraversion, agreeableness, conscientiousness, emotional stability, and openness to experience (DeNeve & Cooper, 1998; McCrae & Costa, 1999). Lee and Song (2017) found that women who experienced sexual abuse early in their life had a lower level of emotional stability than their counterparts. They also found that the effects of early experiences of sexual abuse could leave a long-lasting effect and influence social and psychological development, which can affect personality traits. Bosmans et al. (2015) found that emotional stability strongly predicted the ability to cope effectively with life challenges after a traumatic event.

As a result of diverse personal resources, stress appraisals, and coping processes, the psychological symptoms that were experienced vary widely (Lazarus, 1990). A focus on everyday events and stressors can be useful in understanding how individuals cope after a traumatic event. Some individuals react more strongly and some do better after experiencing a traumatic event. The meaning constructed by a person about what is happening is crucial to the arousal of stress reactions (Lazarus, 1999, 2006).

The theoretical framework of the study draws heavily on the writings of Lazarus (1999, 2006). This implies that the interaction of antecedent personal factors such as sex, age, and personality, and contextual factors such as socioeconomic conditions, shape the meaning that an individual ascribes to a situation, their coping effects, and their emotional conditions.

The aim of the present study was to compare the characteristics of two groups of female survivors of sexual abuse, one group with a lower indication of PTSD (L-PTSD) and one group with a higher indication of PTSD (H-PTSD). We hypothesized that, among women with a history of sexual abuse, the H-PTSD group would be associated with more daily hassles, fewer daily uplifts, and more use of maladaptive coping strategies. We also hypothesized that women in the H-PTSD group would report more severe sexual victimization, less resourceful socioeconomic conditions, and a lower level of emotional stability.

## Method

### Sampling

The criteria for participation were being female, aged 18 years or older, having experienced sexual abuse, and speaking Norwegian. The participants in this study were recruited from support centers for survivors of incest and sexual abuse (SMISOs) in Norway. The centers receive central and local government funding and are primarily a self-help service for adults who have been subjected to sexual abuse. According to numbers from the Norwegian Directorate for Children, Youth and Family Affairs (2019), 60% of the female survivors who contact and use these support centers have experienced sexual abuse as children (aged <14 years) and 19% of the women as adults (aged >18 years). In 2017, the support centers had 1,447 users, both men (20%) and women (80%) (Norwegian Directorate for Children, Youth and Family Affairs, 2019). These centers have expertise in understanding trauma, trauma counseling, and aiding adult survivors with the consequences of their past or more recent trauma experience.

We initially contacted 17 of 22 support centers in Norway, with a geographic variation and variation in the number of users. Nine centers agreed to help in recruiting participants. The data collection lasted from February to October 2018. We lack information about how many women could have participated in the data collection, because some users of SMISOs are anonymous and participation at the center is voluntary. According to the Norwegian Directorate for Children, Youth and Family Affairs (2019), the mean age and nationality of the study participants corresponded fairly well with the overall picture at the SMISO centers.

The study was announced through leaflets, social group meetings at the different centers, and social media (the center’s Facebook site). Those women who wanted to participate received an information letter at the center with details of the goals of the survey, and statements about the voluntary and anonymous nature of participation. Also it detailed that they could withdraw at any time for no reason and with no negative consequences. Those who wanted to participate contacted a designated person at the support center, and received a user name and password to access the self-report questionnaire electronically. Through this, it was not possible to track the web address to the individual participant. Only the recruiter at each center had information about the users who wanted to participate. After some weeks of data collection, we opened up the option to answer the questionnaire on paper and send answers by mail. This option came after feedback, from some of the recruitment personnel, that potential participants were hesitant to send their answers electronically.

The survey took about 30–45 min to complete. The participants were offered an opportunity to talk with a counselor at the center if they needed to, because answering questions about traumatic events could activate thoughts and feelings that could lead to distress.

Fifty-seven women completed the questionnaire: 53% responded electronically and 47% on paper. The mean age of participants was 41.4 years (*SD* = 11.6; range = 19–69). Of the participants 93% were ethnic Norwegian; the remaining 7% were from another Scandinavian country, another European country, or a country outside Europe.

### Measures

The questionnaire was composed of a combination of established, validated scales and newly constructed items based on a recent qualitative study of daily hassles, uplifts, and coping after sexual abuse (Stensvehagen et al., 2019).

#### Demographics and socioeconomic conditions

The demographic and socioeconomic variables included 11 items. The participants were asked to state their birth year, country of birth, number of years living in Norway, level of education, occupational status, number of years working, social network, marital status and whether they had children, current home, and self-assessed economy.

#### Experience of sexual trauma

The participants were asked to specify what kind of sexual trauma they had experienced up to the date of the survey using the categories from the Norwegian Penal Code: unwanted sexual behaviors, unwanted sexual acts, or unwanted intercourse (Penal Code, 2005; Steine et al., 2012). The first category, unwanted sexual behaviors, can be indecent exposure, peeping, or being shown pornographic pictures or highly sexualized behavior without physical contact. The second category, unwanted sexual acts, refers to being touched on the genitals/breasts, being forced to masturbate others, or experiencing repeated sexual intercourse or similar movements against one’s own body. The last category, unwanted intercourse, refers to feeling pressured to undertake sexual intercourse without the presence of violence or threats, forced sexual intercourse by the use of violence or threatening behavior, or experiencing penetration with fingers, objects, or genitals into the vagina or rectum. In addition, the participants were asked questions in relation to the victimization: age when the first sexual abuse occurred, and the sex and age of the abuser, as well as their relationship to him or her.

Many of the questions had several “yes/no” response choices, where more than one could be checked. To illustrate this, the respondents were asked about the sex and age of the abuser(s). The following four response choices were available: (a) adult male, (b) adult female, (c) boy (aged <18 years), and (d) girl (aged <18 years). This question resulted in five possible comparisons: the sum scores of “yes” responses (Mann–Whitney *U*-test) which in this case could range from 0 to 4, and each of the four yes/no response choices (χ^2^ tests). Many of the “yes/no” choices were endorsed by only a few women. Thus, in cells with an expected count <5, Fisher’s exact test was used.

#### Emotional stability

The Single-Item Measures of Personality (SIMP) was used to assess emotional stability (Woods & Hampson, 2005). The items were presented with two dichotomous statements as anchors on a bipolar, 9-point graded line as follows: (1) am sensitive, easy going, happy, and can be tense, to (9) am relaxed, restrained, rarely irritated, and seldom depressed and sad. The questionnaire has shown good convergent and divergent validities (Woods & Hampson, 2005).

#### Daily hassles

Daily hassles were measured using 13 items from the Stress Profile Scale (Setterlind & Larsson, 1995), which was responded to on a 5-point Likert scale from “never” (1) to “very often” (5). In addition, 10 newly constructed items (worries: seven items; irritants: three items) were added based on the qualitative study of experiencing and coping after sexual abuse (Stensvehagen et al., 2019). Examples of new items included are: “Worry of memories from the abuse” and “Irritation at not being taken seriously.” Participants related their responses to experiences over the last month. The following Cronbach’s α coefficients were obtained for the hassle scales: total hassles with 23 items (α = .90); 13 original items (α = .83); and 10 newly constructed hassles (α = .82).

#### Daily uplifts

This was measured with six items from the Stress Profile Scale (Setterlind & Larsson, 1995), with responses on a 5-point Likert scale from “never” (1) to “very often” (5). In addition, seven newly constructed items were added based on the qualitative study of experience of and coping after sexual abuse (Stensvehagen et al., 2019). Examples of new items included: “Joy at having a place where you’re safe” and “Joy at having energy in everyday activities.” Participants related their responses to experiences over the last month. Cronbach’s α for the uplift scales were 13 total uplift items (α = .91), six original items (α = 0.85), and seven newly constructed items (α = .84).

#### Coping strategies

The Brief Coping Orientation to Problems Experienced (COPE) questionnaire (Carver, 1997) was used to measure coping strategies. This is a 28-item questionnaire assessing coping behavior in response to stressful or traumatic situations within the last month. The responses are rated on a 4-point Likert scale, ranging from “I don’t do this at all” (1) to “I do this a lot” (4). Fourteen subscales are generated representing eight adaptive coping strategies (positive reframing, accepting, seeking emotional support, seeking instrumental support, humor, planning, active coping, and religion) and six maladaptive coping strategies (self-distraction, denial, substance use, behavioral disengagement, venting, and self-blame). Subscales were summed to generate one adaptive coping scale with 16 items (α = .78) and one maladaptive coping scale with 12 items (α = .71). In the present study, we used the Norwegian version of the Brief COPE (Kristiansen et al., 2008).

#### Posttraumatic stress

Symptoms of posttraumatic stress were measured using the Norwegian version of the Posttraumatic Stress Disorder Checklist (PCL; Hem et al., 2012). The PCL was first introduced by Weathers et al. (1993) and it is one of the most frequently used self-reported measures on indications for PTSD. The PCL is a 17-item, self-administered questionnaire that assesses PTSD symptoms from the *Diagnostic and Statistical Manual of Mental Disorders* (4th ed.; *DSM*-IV; APA, 1994) (Blanchard et al., 1996). Participants rated how much they were bothered by each symptom over the past month, using a 5-point Likert scale, ranging from “not at all” (1) to “extremely” (5). The PCL score can range from 17 to 85, with higher values indicating increased severity (α = .89) (Hem et al., 2012).

### Statistical Analysis

We conducted the statistical analyses using the Statistical Package for Social Sciences (SPSS) version 24. Summary indices were computed on all the aforementioned instruments, except for the emotional stability scale (SIMP). This was carried out by adding the raw scores of the items belonging to a scale and dividing by the number of items. An exception was the PCL scale when the raw sum was used. Conventional descriptive statistics were computed and subgroup comparisons were performed using χ^2^ tests, including Fisher’s exact test, Kruskal–Wallis one-way analysis of variance (ANOVA) by ranks, and Mann–Whitney *U*-tests. Statistical significance was assumed at *p* < .05. Bonferroni correction was calculated to counteract multiple comparisons. In addition, Cohen’s *d* (Cohen, 1988) values were computed to assess effect size.

### Ethics

The present study was carried out adhering to the ethical principles contained in the Declaration of Helsinki (World Medical Association, 2013), and was approved by the Norwegian Center for Research Data (December 15, 2017, 47,920/AGL).

## Results

### Defining a Lower, Mid, and Higher PTSD Group

Participants with scores in the lowest third on the PTSD scale formed the L-PTSD group (*n* = 19*).* They had a mean score of 40.37 (*SD* = 6.30). The mid-third group (M-PTSD, *n* =18) had a mean score of 53.61 (*SD* = 4.00). Participants with scores in the highest third on the PTSD scale formed the H-PTSD group (*n* = 20). Their mean score on the PTSD scale was 72.45 (*SD* = 5.96). Initially, all subgroup comparisons were made by including all three subgroups (χ^2^ tests and Kruskal–Wallis ANOVA). However, as the subgroup comparisons involving the L-PTSD and the H-PTSD groups show the most clear-cut results, the remaining part of this section is based on these two groups.

In the L-PTSD group, all the women scored lower (from 22 to 46) than the cut-off (≤50), indicating PTSD (Weathers et al., 1993). In the H-PTSD group, all the women scored higher (from 61 to 79) than the cut-off (≤50), indicating PTSD. Linear trends were noted on all hassle, uplift, and coping scales, showing that the L-PTSD group scored most favorably and the H-PTSD group least favorably.

### Demographic and Socioeconomic Characteristics

The mean age of the two groups differed by 2 years (H-PTSD: mean age 39.3 years [*SD* = 10.82], range 20–59 years; L-PTSD: mean age 40.5 years [*SD* = 11.42], range 23–69 years). This difference was not statistically significant. Most of the women in both groups were born in Norway (L-PTSD: *n* = 17; H-PTSD: *n* = 19), and most had lived in Norway for more than 6 years (L-PTSD: *n* = 18; H-PTSD: *n* = 20). The remaining demographically and socioeconomically related data are based on eight questions (Table 1). Of these, only one comparison between the H-PTSD and L-PTSD groups was statistically significant. The educational level showed a significant difference between the two groups (*p* = .013). The L-PTSD group had, overall, the highest educational level, with most reporting having finished college/university. In the H-PTSD group, most had high school as their highest educational level. However, after Bonferroni correction, due to the high number of comparisons, no significant differences remained.

**Table 1.** Demographic and Socioeconomic Comparison of L-PTSD and H-PTSD Subgroups.

Background Data^a^	L-PTSD (*n* = 19) *n*	H-PTSD (*n* = 20) *n*	Pearson’s χ^2^	*df*	*p*
Highest educational level			8.70	2	.013
Primary school	7	4			
High school	2	11			
College/University	10	5			
Occupational status			4.98	2	.083
Employed^b^	9	3			
Unemployed^c^	5	10			
Work assessment allowance^d^	5	7			
Number of years working			0.89	1	.345
≤3	7	5			
>3	10	14			
Social network			1.25	1	.264
≤2 close relationships	8	12			
>2 close relationships	11	8			
Marital status			0.03	1	.855
Single	11	11			
Married/partner	8	9			
Children			3.09	1	.079
Yes	7	13			
No	12	7			
Current home			0.04	1	.839
Rents an apartment or house	12	12			
Owns an apartment or house	7	8			
Self-assessed economy			1.37	1	.243
Not so good	12	16			
Good/Very good	7	4			

*Note.* L-PTSD = lowest third on PTSD symptom scale; PTSD = posttraumatic stress disorder; H-PTSD = highest third on PTSD symptom scale.

a Categorical data. ^b^Employed: full-time or part-time employment. ^c^Unemployed: student, retired, on sick leave, or on disability benefit. ^d^A work assessment allowance allows a person who is impaired by at least 50% to have an income in periods during which they are ill or injured and need assistance from the Norwegian Labor and Welfare Administration to return to work (Norwegian Labour and Welfare Administration, 2020).

### Sexual Trauma–Related Characteristics

Table 2 shows that, when counting all the “yes” answers, in legal terms, all the women in both groups had experienced the most severe type of sexual abuse.

**Table 2.** Trauma-Related Data Comparison of the L-PTSD and H-PTSD Subgroups.

Sexual Trauma–Related Data	L-PTSD		H-PTSD		Mann–Whitney	*p*	Cohen’s *d*
	(*n* = 19)		(*n* = 20)				
	*M*	*SD*	*M*	*SD*	*U* test		
Sexual abuse (total)^a^	5.11	2.11	5.40	2.16	171.00	.607	0.14
Unwanted sexual behaviors^b^	0.63	0.90	0.80	0.83	164.50	.478	0.20
Unwanted sexual acts^c^	2.16	1.17	2.55	1.19	146.50	.224	0.33
Unwanted intercourse^d^	2.00	0.94	2.00	1.03	187.00	.945	0.00

*Note.* Cohen’s *d* measures the effect size: *d* = 0.2 (small); *d* = 0.5 (medium); *d* = 0.8 (large) (Cohen, 1988). L-PTSD = lowest third on PTSD symptom scale; PTSD = posttraumatic stress disorder; H-PTSD = highest third on PTSD symptom scale.

a Sexual abuse (total) includes all possible response choices included in the three subcategories unwanted sexual behavior, unwanted sexual acts and unwanted intercourse (the scale could range from 0 to10). ^b^Unwanted sexual behaviors include the following possible response choices (the scale could range from 0 to 4): indecent exposure/peeping; being shown pornographic pictures; being exposed to highly sexualized behavior without physical contact. ^c^Unwanted sexual acts include the following possible response choices (the scale could range from 0 to 3): being touched on genitals/breasts; being forced to masturbate others; experiencing repeated sexual intercourse or similar movements against one’s own body. ^d^Unwanted intercourse includes the following possible response choices (the scale could range from 0 to 3): feeling pressured to sexual intercourse without the presence of violence or threats; forced sexual intercourse by the use of violence or threatening behavior; experiencing penetration with fingers, objects, or genitals in the vagina or rectum.

Most of the women in both groups had experienced sexual abuse at an early age (age <7 years); the L-PTSD: *n* = 11 (*M* = 1.79, *SD* = 0.25) and the H-PTSD: *n* = 12 (*M* = 1.55, *SD* = 0.17). First sexual trauma after the age of 18 years was reported only in the L-PTSD group (*n* = 2).

In both groups, most of the women knew their offender: L-PTSD: *n* = 18; H-PTSD: *n* = 17. Some of the women also reported being sexually abused by an unknown offender: L-PTSD: *n* = 6; H-PTSD: *n* = 5. The offender was mostly an adult male (L-PTSD: *n* = 16; H-PTSD: *n* = 20). In some cases the offender was aged <18 years (L-PTSD: *n* = 8; H-PTSD: *n* = 7). Some had also had their experience with adult female offenders (L-PTSD: *n* = 3; H-PTSD: *n* = 2). In a few cases the offender was female and aged <18 years (L-PTSD: *n* = 1; H-PTSD: *n* = 2). The differences between the two groups were not statistically significant on any of the trauma-related questions.

### Emotional Stability

A Mann–Whitney *U*-test indicated that emotional stability was greater for women in the L-PTSD group, than for those in the H-PTSD group (Table 3).

**Table 3.** Emotional Stability, Hassles, Uplifts, and Coping Comparison of the L-PTSD and H-PTSD Subgroups.

Variable	L-PTSD (*n* = 19)		H-PTSD (*n* = 20)		Mann–Whitney *U* test	*df*	*p*	Cohen’s *d*
	*M*	*SD*	*M*	*SD*				
**Emotional stability** ^a^	3.84	1.21	2.55	1.85	8.93	2	.004	0.82
**Hassles (total)** ^b^	2.95	0.49	3.91	0.51	25.98	2	**<.001**	1.93
*Worries (total)*	3.04	0.58	4.23	0.53	27.43	2	**<.001**	2.13
Worries original scale	3.20	0.58	4.40	0.50	26.00	2	**<.001**	2.20
Worries newly constructed scale	2.90	0.68	4.08	0.69	22.03	2	**<.001**	1.72
*Irritant (total)*	2.83	0.54	3.50	0.68	11.99	2	.**001**	1.09
Irritant original scale	2.58	0.67	3.09	0.70	5.69	2	.030	0.75
Irritant newly constructed scale	3.40	0.65	4.43	0.81	17.52	2	**<.001**	1.40
**Uplifts (total)** ^c^	3.38	0.56	2.83	0.76	8.90	2	.022	0.83
Uplifts original scale	3.44	0.66	3.09	0.81	6.91	2	.020	0.47
Uplifts newly constructed scale	3.34	0.57	2.61	0.76	10.15	2	.004	1.08
**Coping** ^d^								
Adaptive coping	2.59	0.47	2.57	0.40	0.92	2	.967	0.05
Maladaptive coping	1.82	0.36	2.59	0.47	21.75	2	**<.001**	1.84

*Note.* Text in bold refers to significance after Bonferroni correction. Cohen’s *d* measures the effect size: *d* = 0.2 (small); *d* = 0.5 (medium); *d* = 0.8 (large) (Cohen, 1988). L-PTSD = lowest third on PTSD symptom scale; PTSD = posttraumatic stress disorder; H-PTSD = highest third on PTSD symptom scale.

a Emotional stability: Single Item Measure of Personality (SIMP): a single-item measure presented as two dichotomous statements. The item was measured on a bipolar 9-point graded line. ^b^Stress profile from Setterlind and Larsson (1995). Hassles measured on a 5-point Likert scale from “never” (1) to “very often” (5). Hassles (total) includes worries (total) and irritant (total) from the original scale and newly constructed scale (Stensvehagen et al., 2019). ^c^Stress profile from Setterlind and Larsson (1995). Uplifts measured on a 5-point Likert scale from “never” (1) to “very often” (5). Uplifts (total) includes items from the original scale and newly constructed scale (Stensvehagen et al., 2019). ^d^Coping is computed from items in the Brief COPE Questionnaire (Carver, 1997). Ratings on a 4-point Likert scale, “I don’t do this at all” (1) to “I do this a lot” (4). The scale is divided into adaptive and maladaptive coping strategies, according to Carver (1997).

### Daily Hassles, Uplifts, and Coping

The hassle-related data consist of seven indices. As Table 3 shows, there were significant differences between the groups on all these scales. The L-PTSD scored most favorably on all.

The uplift-related data consist of three indices. Of these, “uplift total” and “uplift newly constructed” showed statistically significant differences between the H-PTSD and the L-PTSD groups (Table 3). Thus, the H-PTSD group reported lower frequencies of daily uplifts over the last month.

The coping-related data consist of two indices. The results show that there were significant differences in maladaptive coping strategies (Table 3). In the L-PTSD group, we found more adaptive coping strategies and in the H-PTSD group significantly more maladaptive coping strategies. In 11 of 12 comparisons on the hassle, uplift, and coping scales the results showed significant differences between the L-PTSD and the H-PTSD groups. After Bonferroni correction, seven of the differences remained significant.

## Discussion

In the present study, we compared two groups of female survivors of sexual abuse with either a higher or a lower total score indicating PTSD. We compared the two groups on trauma-related, demographic and socioeconomic conditions, personality traits, and the hassle, uplift, and coping variables. The present study provides support for the first hypothesis that higher levels of PTSD symptoms were associated with more daily hassles, fewer daily uplifts, and more maladaptive coping strategies.

The differences between the L-PTSD and H-PTSD groups on the hassle and uplift scales are in the expected direction, showing that women who reported high levels indicating PTSD also reported more daily hassles, which is in line with the limited existing research (McGuigan & Middlemiss, 2005; Thakkar & McCanne, 2000). This is in agreement with Walsh et al. (2010) who found, in their review of adults coping after sexual abuse, that survivors reported a variety of coping strategies, which evolved and changed as the phases of coping with trauma changed.

We also hypothesized that women with higher levels of PTSD symptoms would report more severe sexual victimization, less resourceful socioeconomic conditions, and a lower level of emotional stability. The hypothesis was unsupported, except for emotional stability. Given the high number of nonsignificant differences between the L-PTSD and the H-PTSD groups, the conclusion is that severity of sexual abuse and socioeconomic conditions had little or no effect on the current indication of PTSD.

The lack of differences on the sexual abuse scales could be due to a ceiling effect. The two groups in this study were selected from a sample in which all reported severe trauma experiences. Most (17 in the L-PTSD group and 20 in the H-PTSD group) of the women had experienced sexual abuse before the age of 18 years, many before the age of 7 years (11 women in the L-PTSD group and 12 women in the H-PTSD group). Research has shown that childhood sexual abuse influences a number of adult developmental outcomes such as mental disorders, psychological wellbeing, sexual risk taking, physical health, and socioeconomic wellbeing (Fergusson et al., 2013). Most of the women in the study group had experienced unwanted intercourse. As almost everyone had experienced the most serious kind of abuse, our data do not give an answer on whether the degree of severity of the abuse is associated with high or low levels indicating PTSD.

The results concerning demographic and socioeconomic data revealed marginal differences between the two groups. However, the participants all attended a support center for survivors of incest and sexual abuse. These centers may contribute to a different kind of social network, through activities arranged at the center. This can be an important contribution that also safeguards some form of social network, which is an important contributor to recovery (Stensvehagen et al., 2019). A downside to this is if the centers, over time, become the only link to social support and a substitute for family and friends. In a summary of the demographic and socioeconomic factors, we do not rule out that these factors may have an effect on PTSD symptoms after sexual abuse, because this has been shown in previous research (Gekker et al., 2018). However, this was not the case in the present study of severely victimized women. Once again, one explanation is that the severity of the sexual abuse outweighed the potential impact of demographic and socioeconomic factors.

According to Almeida (2005), personality traits are one of several factors that influence resilience and vulnerability to daily stressors. In the present study, we found that there was a significant difference in emotional stability between the two groups. Those with high levels indicating PTSD scored low on emotional stability. Lee and Song (2017) also found that low emotional stability was significantly associated with childhood abuse (physical, emotional, and sexual). Based on this cross-sectional study, it cannot be concluded whether either the sexual abuse caused a lower emotional stability or a lower emotional stability resulted in higher symptom reporting. Drawing on a more general psychological framework, it could be argued that there is a bidirectional causality, but that the main influence route would be that a lower emotional stability would contribute to an increased vulnerability to highly stressful events (Lazarus, 1999, 2006).

The novelty of the present study lies in the results that indicate that PTSD symptom intensity among severely sexually abused women was strongly related to a high degree of perceived daily hassles, low amounts of daily uplifts, a lot of use of maladaptive coping strategies, and a low level of emotional stability, combined with little or no relationship to demographic and socioeconomic factors and severity of the sexual abuse. The results on the hassle, uplift, and coping scales are potentially interesting from an interventional point of view. Major life events such as sexual abuse may be out of control for the afflicted woman. Appraisal of and coping with everyday events, however, can be affected and offer interesting possibilities for interventions directed at the survivors, their significant others, and professional helpers.

We now turn to some limitations of the present study. As with all cross-sectional data, we can access only the association and cannot make assumptions about the causal directions of our variables. Nonresponse is a particular problem affecting cross-sectional studies and can result in bias of the measures of outcome. Many women never talk about their trauma, suffer in silence, and are difficult to recruit (McTavish et al., 2019). In the present study, we recruited women attending a support center for survivors of incest and sexual abuse, which indicates that our sample is a selected group receiving support and counseling. Recruitment of a more representative sample may have revealed other answers to our hypothesis.

Our sample size was small and this limits statistical power to detect significant differences between the H-PTSD and L-PTSD groups and the generalizability of our findings. To correct for Type 1 error, resulting from multiple analyses and a small sample, we calculated Bonferroni correction. In addition, we calculated effect size when measuring two groups through the use of Cohen’s *d.* The results in the present study, which were significant before Bonferrori correction and not significant after, showed a medium (0.5) or large (0.8) effect size when using Cohen’s *d*. This indicates that, even though the sample size was small, these results still show that there were differences between the L-PTSD and the H-PTSD groups.

The centers, from which we recruited participants, have in recent years been subjected to several requests for research participation, and this could be one explanation for a reluctance to participate. For some it may have been too demanding to answer the questionnaire. In addition, shame, guilt, and threats from others can be contributing factors to women choosing not to participate (Kennedy & Prock, 2018; Reitsema & Grietens, 2016).

We know little about those who chose not to respond to the survey. The overall feedback from the recruiters at the support centers was positive, and they reported that the feedback from participants was that the questions were perceived as meaningful. Some skepticism was reported toward answering the questions electronically. Although there was the opportunity to participate through answering the questions both electronically and on paper, participation was still low. In retrospect, we think that, by using only a questionnaire on paper from the start of the data collection, possibly a larger number of women would have answered the questionnaire in our study.

The participants in this study were a selected group of mostly Norwegian women, so our results are not transferrable to women from other countries or cultures.

We also asked participants about past exposure, even though the period addressed in the questionnaire was in the last month. Still, this could result in a recall bias that may have influenced our results (Polit & Beck, 2017).

It is a known fact that survey respondents may have a tendency to answer questions in a manner viewed favorably by others, and that this can lead to social desirability bias (Polit & Beck, 2017). Whether the participants in this study overreported “good behavior” or underreported undesirable behavior is difficult to determine. The feedback from those responsible for recruiting participants at the centers was that the survey had been perceived as relevant, although somewhat time-consuming to respond to.

A strong point of our sample is that we have recruited a group of severely traumatized women. It is not certain that we would have managed to reach this group using other recruitment methods. Finally, the present study is one of very few studies that have developed and tested more context-specific questions on hassles and uplifts in a group of female survivors of sexual abuse.

### Research Implications

Future research would benefit from the development of coping instruments, which incorporate concepts hypothesized to be specific to trauma, as suggested by Walsh et al. (2010). Comparison of groups of female survivors of sexual trauma should be replicated in larger populations across different cultures. It is important to acknowledge that, even though our focus has been on female survivors of sexual abuse, it is important to focus on male survivors. It would be interesting to carry out a similar study with male survivors, to find out whether there are gender differences or similarities with regard to daily hassles, uplifts, and use of coping strategies.

## Conclusion

In the present study we compared groups of female survivors of sexual abuse with high (H-PTSD) and low (L-PTSD) levels indicating PTSD. All of the women had experienced severe sexual abuse. The study provides support for the hypothesis that the women with more symptoms indicating PTSD (H-PTSD) reported having significantly more daily hassles and fewer daily uplifts, and used more maladaptive coping strategies. The L-PTSD group reported more emotional stability, fewer daily hassles, and more uplifts, and used more adaptive coping strategies. Few differences were found between the H-PTSD and the L-PTSD groups with regard to severity of sexual abuse and socioeconomic conditions. Major life events such as sexual abuse may be out of control for the afflicted victim. Appraisal and coping with everyday events can be affected and offer interesting possibilities for interventions.
